# Combined repetitive facilitative exercise under continuous neuromuscular electrical stimulation and task-oriented training for hemiplegic upper extremity during convalescent phase after stroke: before-and-after feasibility trial

**DOI:** 10.3389/fneur.2024.1356732

**Published:** 2024-02-22

**Authors:** Koya Fujimoto, Makoto Ueno, Seiji Etoh, Megumi Shimodozono

**Affiliations:** ^1^Department of Rehabilitation, Kirishima Sugiyasu Hospital, Kirishima, Kagoshima, Japan; ^2^Department of Rehabilitation and Physical Medicine, Kagoshima University Graduate School of Medical and Dental Sciences, Kagoshima, Japan

**Keywords:** stroke, rehabilitation, hemiparesis, task-oriented repetitive facilitative exercise, RFE, NMES, aid for decision-making in occupation choice, ADOC

## Abstract

**Introduction:**

Whereas repetitive facilitative exercise (RFE) affects primarily recovery of motor impairment after stroke, task-oriented training (TOT) focuses on facilitating daily use of the affected upper extremity. However, feasibility of combined RFE and TOT has not been reported. We originated “task-oriented RFE,” as a new combination therapy for patients with hemiplegic upper extremity after subacute stroke, to examine its feasibility in convalescent rehabilitation wards.

**Methods:**

This is a before-and-after pilot study. Eight patients with hemiplegic upper extremity after subacute stroke received the task-oriented RFE program for 6 weeks at 80 min per day (20–60 min of TOT applied after 60–20 min of RFE under continuous neuromuscular electrical stimulation) in a convalescent rehabilitation ward. In the current program, we introduced the Aid for Decision-making in Occupation Choice (ADOC) iPad application as a goal-setting method for determining tasks. Feasibility was assessed with adherence to the protocol, adverse events in response to the intervention, and preliminary efficacy. Motor functions, amount of use and quality of movement in the hemiparetic upper extremity, and satisfaction of the patients were evaluated with Fugl-Meyer Assessment (FMA), the Action Research Arm Test (ARAT), the motor activity log (MAL) for the amount of use (AOU) and quality of movement (QOM) of the paralyzed hand, and ADOC.

**Results:**

All participants accomplished the program, which was implemented as originally planned; neither nonattendance nor an adverse event occurred during the study. Favorable outcomes were obtained with all measures; mean changes in FMA, ARAT in the dominant hand, MAL-AOU, and MAL-QOM were greater than minimal clinically important differences. Mean changes in ADOC were greater than the minimal detectable change.

**Discussion:**

The task-oriented RFE program was safe, well-tolerated, beneficial, and feasible within 80 min a day of occupational therapy, which means also within the procedural constraints of the Japanese health insurance system during the convalescent phase. Future studies are warranted to examine whether combined RFE and TOT enhances the efficacies of each program alone.

## 1 Introduction

Declining motor function associated with paralyzed upper extremity is an important contributor to reduced quality of life among patients who have suffered a stroke (1). It has been estimated that fewer than 15% of patients recover fully from the initial paralysis after a stroke (2). In addition, decreased frequency of use of a paralyzed hand after stroke leads to atrophy of the motor-related cortical area in the damaged hemisphere. As a result, motor output from the brain to the paralyzed hand is further reduced and the paralyzed hand is used less and less. This vicious circle would result in continued non-use of the paralyzed hand, and it is advocated to use the term “learned non-use” (3). Preventing learned non-use in inpatient rehabilitation might result in improved quality of life after discharge. To achieve this goal, we need to provide a rehabilitation program that encourages continued mindfulness of using the paralyzed hand even after discharge from the hospital. Because the medical insurance system generally limits length of stay in the hospital or number of daily sessions for rehabilitation treatment, we have to develop a more effective and efficient rehabilitation program to restore function of the paralyzed hand and to increase frequency of its use.

Reconstruction and reinforcement of neural circuits follow the Hebbian theory: (1) neural circuits that repeatedly transmit excitement are reinforced; (2) neural circuits that are excited at the same time tend to join together (4), and it is important to selectively and repeatedly transmit excitement to the neural pathways (5). To apply this concept to rehabilitation for hemiplegia after stroke, Kawahira et al. (5–7) developed a repetitive facilitative exercise (RFE), in which manual stretch reflexes or skin-muscular reflexes are simultaneously elicited to the specific area (joints) the patient intends to move. RFE aims to reconstruct and reinforce the motor descending tract by realizing the patient’s intended movement and intensively repeating it about 100 times in a few minutes per movement pattern. It has been reported that RFE improves not only motor control but also motor function (ability to manipulate objects) of the hemiplegic limb in addition to enhancing quality of life after stroke (6, 8, 9). In addition, RFE under continuous neuromuscular electrical stimulation (RFE-under-cNMES) has been reported to have greater efficacy than conventional treatment for severe paralysis during the convalescent period after stroke (10, 11). Furthermore, NMES might have an additional effect in the combined use of repetitive transcranial magnetic stimulation and RFE for recovering the ability to manipulate objects and overcome spasticity in the affected upper extremity (12). Indeed, according to a systematic review (13), using electrical stimulation improves motor control and the ability to manipulate objects of the affected upper extremity in comparison with placebo controls. Although improvements in physical function with RFE were reported, there are few studies that report the impact of RFE on increasing use of the affected limb during daily life activities.

On the other hand, a representative rehabilitation treatment that focuses on targeted movements and activities is task-oriented training (TOT) (14). It is recommended as Class I (Level of Evidence A) in the guidelines for adult stroke rehabilitation (15). TOT is important to meet 15 standard components of therapy; in particular, “random and distributed practice, feedback, and clear functional goals” are important for upper extremity functional training after stroke (14). Constraint-induced movement therapy (CIMT) is an evidenced neurorehabilitation that incorporates TOT as one of its components (16). CIMT is a treatment method for improving motor functions or daily use of the affected upper extremity by restraining the non-paralyzed side with a mitten or an arm sling for 6 h a day and forcing the patient to use the paralyzed upper extremity (17). Inclusion criteria for CIMT are reported to be “at least 20° of wrist extension and at least 10° of active extension of each metacarpophalangeal and interphalangeal joint of all digits” (18), which means that patients with moderate to severe paralysis may not be able to participate in or continue a CIMT program. In addition, restraint of the unaffected hand during the subacute phase of stroke may be unnecessary because the effects of intensive bimanual training are reported to be equivalent to a modified version of CIMT (19).

Therefore, we devised a rehabilitation program “task-oriented RFE” that combines “RFE under cNMES” and “TOT” within daily sessions to take advantage of the benefits of each (20). Namely, “RFE under cNMES” facilitates recovery of motor control or motor function in the hemiplegic upper extremity in a relatively short period of time. On the other hand, “TOT” facilitates frequent use of, and improvement in, the hemiparetic side of the upper extremity in real daily life. We thought that the combination could enhance recovery of the paralyzed upper extremity or hand in a short time and efficiently.

In TOT, task setting is also considered important (14). Thus, we introduced Aid for Decision-making in Occupation Choice (ADOC) (21, 22) in “task-oriented RFE” as a goal-setting method for determining tasks (21). ADOC is an iPad application in which the occupational therapist selects important tasks from 95 illustrations of daily living tasks and collaborates with the patient to set goals (22). The use of illustrations supports recall of important activities for oneself better than verbal interviews alone (21, 23). ADOC can also objectively show changes in satisfaction by rating the satisfaction of determined goals.

In this pilot study, a 6-week, 80 min/day program of task-oriented RFE was introduced to patients who had upper extremity hemiplegia after subacute stroke to examine its feasibility during Japanese inpatient rehabilitation in the convalescent phase, which is typically from approximately 1 to 6 months after onset (24). Assessment included patient attendance, acceptability, implementation fidelity, and any adverse effect of the intervention. Additionally, we explored its preliminary efficacy.

## 2 Materials and methods

### 2.1 Subjects

Inclusion criteria of this study were: (1) a new, single stroke with 28–60 days duration, confirmed with a brain CT or MRI; (2) ability to pick up and drop one sheet of tissue paper (25); (3) stable sitting position; (4) Brunnstrom recovery stage ≥3 in the upper extremity; and (5) clinically stable medical state. Exclusion criteria were: (1) shoulder pain; (2) severe cognitive dysfunction that would interfere with understanding instructions from the physician or therapist; and (3) contraindication for electrical stimulation, such as a pacemaker implant.

Subjects were recruited from among inpatients at Kirishima Sugiyasu Hospital in Japan, from April 2020 to July 2021. Six men and 2 women met the inclusion criteria. Characteristics of participants are presented in Table 1. Mean age was 73.6 (SD 13.2, range 47–91). This study was performed in accordance with the principles stated in the Declaration of Helsinki. All participants provided written informed consent, and the study was approved by the ethics committee of Kirishima Sugiyasu Hospital (20001) and was retrospectively registered with the UMIN Clinical Trial Registry (UMIN000051067).

**Table 1:** Participant characteristics at baseline.

*Patients*	A	B	C	D	E	F	G	H	Mean (SD)
Age (years)	63	47	81	76	76	91	76	79	73.6 (13.2)
Sex	M	F	F	M	F	M	M	M	
Type of stroke	I	H	I	I	H	I	H	H	
Site of lesion	Put	Put	CR	CR	Put	CR	Thala	Put	
Side of motor deficit	L	R	L	R	R	L	R	R	
Dominant hand	R	R	R	R	R	R	R	R	
Time from stroke onset (days)	29	54	30	40	57	47	38	45	42.5 (10.2)
Brunnstrom stage (upper limb/hand)	III/IV	III/III	III/IV	IV/IV	IV/IV	IV/IV	IV/IV	III/III	
FMA-upper extremity	20	15	31	34	31	41	40	17	28.6 (10.1)
ARAT	10	6	9	26	13	30	32	11	17.1 (10.4)
MAL-A	0.50	0.23	0.33	0.30	0.25	1.16	1.55	0.00	0.54 (0.53)
MAL-Q	0.60	0.15	0.33	0.38	0.16	1.00	1.75	0.00	0.55 (0.58)
ADOC	1.50	1.00	1.25	1.50	1.66	1.33	1.50	1.50	1.41 (0.21)

SD, standard deviation; M, male; F, female; I, infarction; H, hemorrhage; Put, Putamen; CR, Corona radiata; Thala, Thalamus; L, left; R, right; BRS, Brunnstrom recovery stage; FMA, Fugl-Meyer Assessment; ARAT, action research arm test; MAL-A, motor activity log - amount of use; MAL-Q, motor activity log - quality of movement; ADOC, aid for decision-making in occupation choice.

### 2.2 Study design

A before-and-after pilot design was adopted to investigate feasibility of, and outcomes produced by, the task-oriented RFE. Participants received task-oriented RFE for 6 weeks at 80 min per day, within the limit of what is provided for by the Japanese health insurance system during the convalescent phase—i.e. within the period of time (150 days after hospitalization) and within the total rehabilitation time per day (180 min, including occupational therapy, physical therapy and speech language hearing therapy) that are covered by insurance.

### 2.3 Intervention

**Figure 1:**
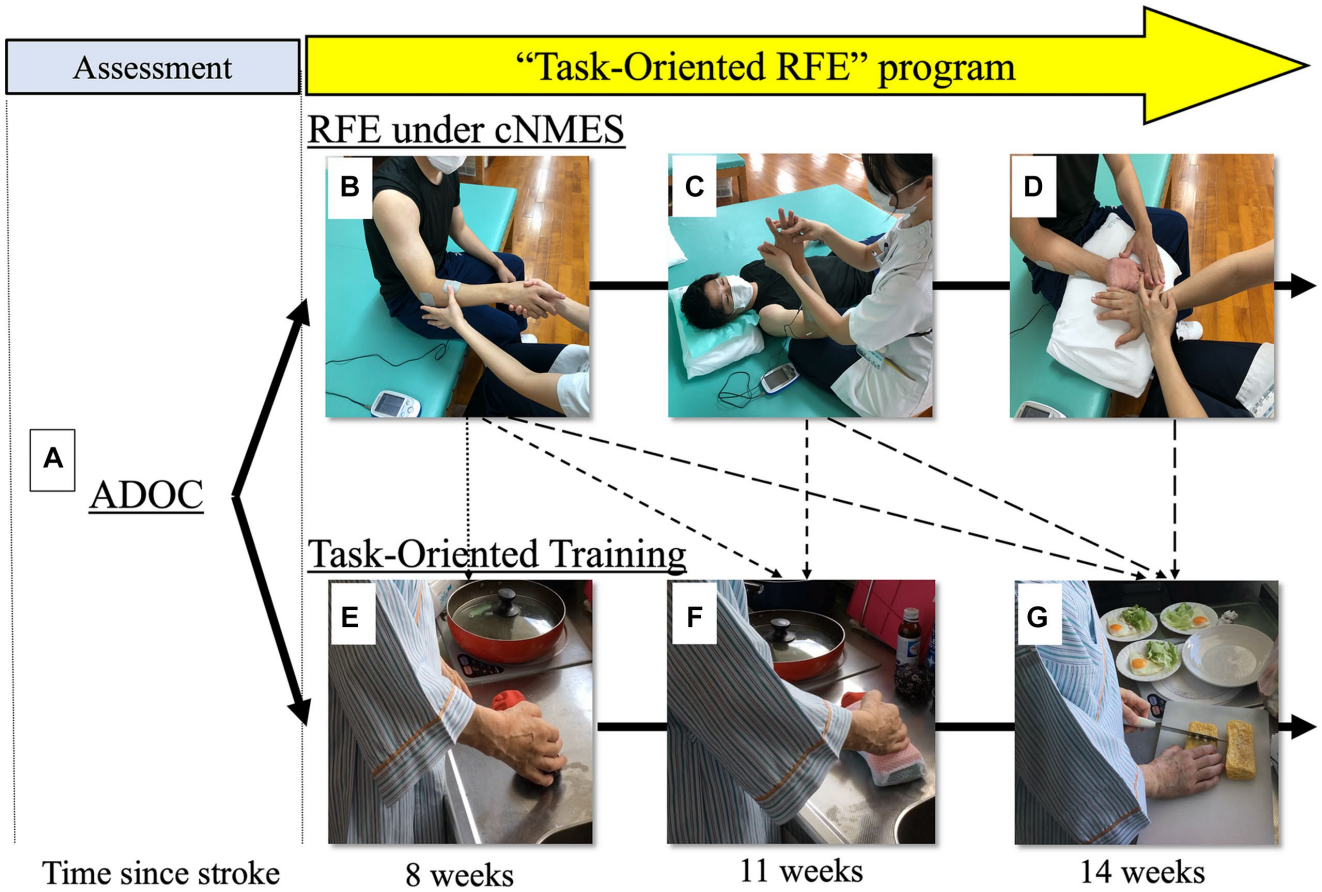
Protocol for task-oriented repetitive facilitative exercise (task-oriented RFE). Bold arrows show step-by-step progression of the “task-oriented RFE” program according to stage of recovery from hemiplegia (the example is from a representative patient, participant E, during about 6 weeks; the subject in the photographs [upper row] is a model). The program consists of “repetitive facilitative exercise under continuous neuromuscular electrical stimulation” (RFE under cNMES, the progression in the upper row) and task-oriented training (TOT, the progression in the lower row). Dotted arrows show correspondence between “RFE pattern” and TOT. First of all, the patient and therapist set as a goal “planning and preparing meals” with ADOC **(A)**. To be able to cut an object during cooking, the patient has to be able to fix an object by “forearm pronation” with her paretic right upper extremity. So, in the daily session, after the therapist chose/performed “forearm pronation and supination pattern” of RFE for the patient **(B)**, the patient participated in TOT including fixing “simulated food” with her forearm in the pronation position **(E)**. After 3 weeks the patient became able to fix the object with her forearm in the pronation position **(F)**, so she was challenged to hold an object with her forearm in the pronation/wrist in dorsiflexion position. To be able to do this, the therapist chose/performed “wrist dorsiflexion/forearm pronation pattern” **(C)** in addition to “forearm pronation and supination pattern” of RFE. After another 3 weeks, the patient was able to accomplish it, so she was challenged to hold an object with her forearm in the pronation/wrist dorsiflexion/individual finger flexion and extension position **(G)**. To achieve this, the therapist chose/performed “finger extension or flexion pattern” **(D)**, in addition to **(B,C)**. Abbreviations; ADOC, aid for decision-making in occupation choice; RFE under cNMES, repetitive facilitative exercise under continuous neuromuscular electrical stimulation; TOT, task-oriented training.

Task-oriented RFE was conducted by combining RFE-under-cNMES, which mainly focuses on improving motor impairment and motor control, and TOT, which focuses on achieving target activities including manipulation of objects. Figure 1 illustrates the “task-oriented RFE” protocol. Before starting treatment of the upper paretic extremity, ADOC was used to select, set, and share the patient’s treatment goals. In the daily training session, occupational therapists performed RFE-under-cNMES at first, and then TOT. Intervention lasted for a total of 80 min, comprising RFE-under-cNMES (20–60 min) and TOT (20–60 min). Proportion of time allotted to each of the two interventions was determined by each occupational therapist according to degree of paralysis, recovery, and goal achievement based on clinical judgment. For example, in a case of severe paralysis, time spent on RFE accounted for up to 60 min out of the 80-min total. As paralysis improved, time spent on RFE gradually decreased, while time spent on TOT (i.e., training for manipulation of objects) gradually increased up to a maximum of 60 min. Further, progression of RFE was determined by a standardized method with respect to degree of paralysis/recovery (Brunnstrom recovery stage) (7); progression of TOT was determined by each therapist with respect to degree of goal achievement based on clinical judgment.

The intervention period was 5 days/week, for 6 weeks. Treatments were conducted by 3 trained and skilled occupational therapists who had completed an RFE training course held in Japan and had 5 to 9 years of clinical experience including task-oriented training.

ADOC was occasionally used at the beginning of the daily session when the attending occupational therapist felt that the patient needed to modify goals or clarify treatment objectives as the function of the paralyzed limb was regained.

#### 2.3.1 RFE under cNMES

In RFE-under-cNMES to the upper limb and fingers, continuous neuromuscular electrical stimulation (cNMES) was performed during RFE to achieve each target movement with the paralyzed limb and fingers (10).

The theory and training methods of RFE are presented elsewhere (6–8, 10, 26). Briefly, with RFE the therapist elicits a stretch reflex by rapidly stretching the target muscle and synchronizes it with a voluntary contraction to achieve the target movement.

**Figure 2:**
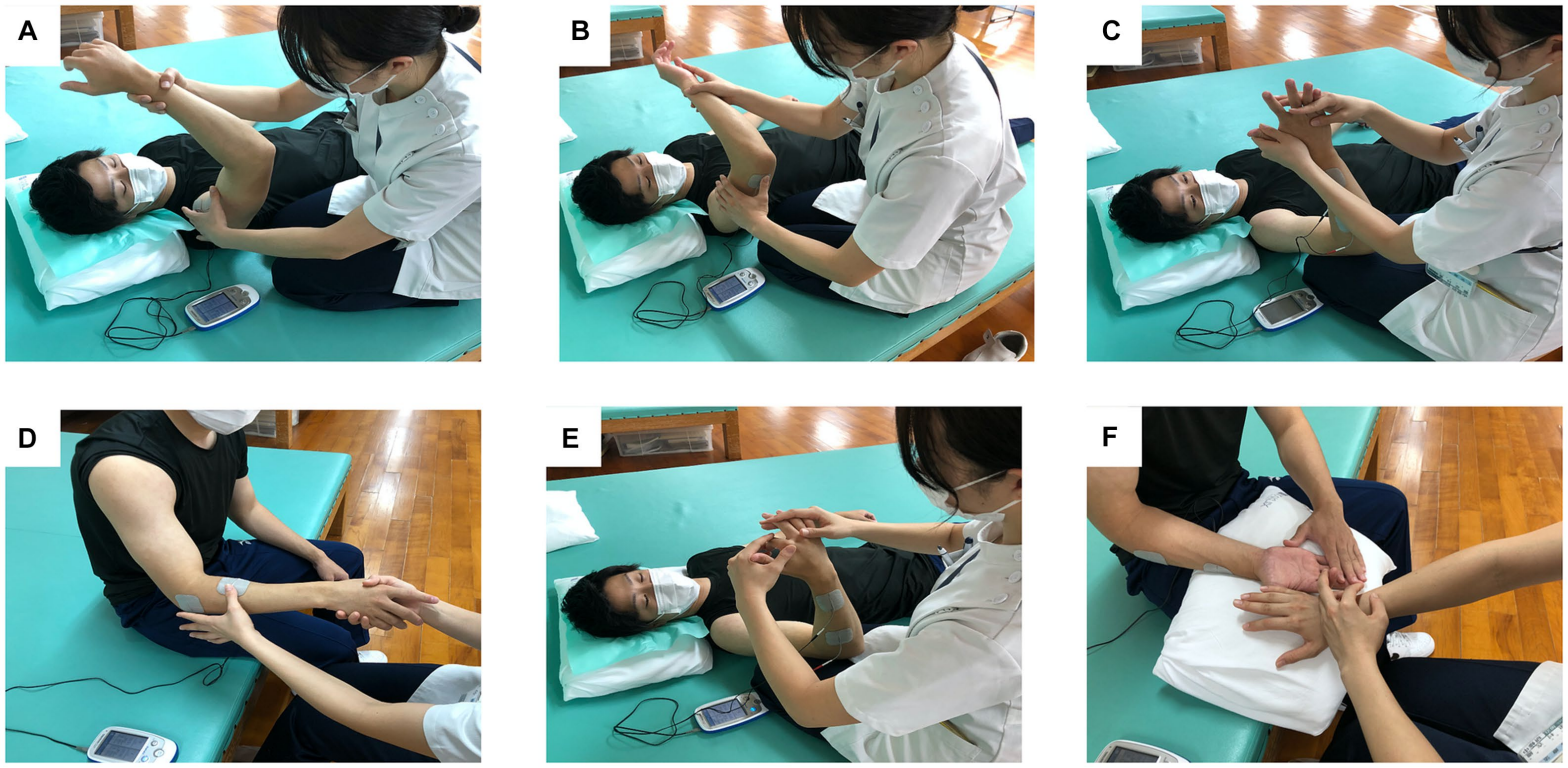
Representative exercise patterns of repetitive facilitative exercise (RFE) under continuous neuromuscular electrical stimulation (cNMES) for a patient with right hemiplegia. The subject shown in the figure is a model. Movement pattern and site of electrical stimulation are as follows: **(A)** shoulder flexion, deltoid muscle; **(B)** elbow flexion and extension, triceps brachii muscle; **(C)** wrist dorsiflexion, extensor carpi ulnaris muscle; **(D)** forearm pronation and supination, supinator muscle; **(E)** finger extension, extensor digitorum muscle; **(F)** finger extension or flexion, extensor digitorum muscle.

There are 15 treatment patterns of RFE, for shoulder, elbow, forearm, hand, and fingers (7). From these, 3 to 8 patterns were selected according to the degree of paralysis and the target movement of the patient. Figure 2 shows 6 typical treatment patterns.

The intervention time was about 20–60 min (as noted above), and the exercise pattern of RFE included 50 to 100 repetitions, depending on the patient’s fatigue level and needs. Additionally, cNMES aims to increase the level of excitation of the target muscle. It facilitates the appearance of a stretch reflex by slightly contracting the target muscle before stretching it, and it also assists muscle contraction during voluntary contraction. The electrical stimulation was a symmetrical biphasic waveform with a pulse width of 200 μs and a frequency of 50 Hz. The electrical current intensity was set so that the target muscle contracted slightly. This low-amplitude NMES was continuously applied to the prime mover (target muscle) during RFE (i.e., cNMES). A portable surface neuromuscular stimulator (ESPURGE, Ito Co., Ltd., Tokyo) was used to apply cNMES.

The electrical stimulation should not cause “passive” joint movement. To induce the stretch reflex, the therapist manually signals the patient at the moment of stretching the target muscle, which results in a sustained contraction of the target muscle at the patient’s will, with the electrical stimulation assisting in that contraction. The motor pattern of hand extension is shown as an example. A pair of surface electrodes is attached to the target muscle, such as the extensor digitorum, and electrical stimulation is applied. The therapist visually confirms that the target muscle is stimulated without exerting force. The stimulation intensity is then reduced and adjusted to achieve a slight contraction.

#### 2.3.2 Task-oriented training

With TOT it is important to (1) perform the practical exercises necessary for the target activity, (2) use objects, and (3) clearly set practical goals that respect the patient’s needs. CIMT generalizes TOT with the concepts of “shaping” and “task practice” (27). In the current TOT program, we take the concept from CIMT, especially shaping and task practice; however, we did not adopt the practice of restraining the unaffected upper extremity with a mitten or glove. Shaping is an approach to achieving motor function and activity goals by encouraging step-by-step adjustments to the exercises. Many tasks involve object manipulation training, such as moving blocks. Task practice is a method focused on improving the performance of such activities. The goal is to enable the patient to perform the target task with the paralyzed hand by using environmental settings and self-help devices.

It is reported that shaping improves motor function, and task practice increases use of the paralyzed upper extremity (28). Training is performed with progressively increasing difficulty suited to the upper-extremity function and physical condition of the patient. In addition, we added to cNMES the extensor digitorum and extensor carpi ulnaris muscles during TOT if wrist dorsiflexion and hand extension were insufficient.

In addition to the occupational therapy including task-oriented RFE, physical therapy and speech language hearing therapy were provided for 40–60 min per day, but with no active intervention for the upper extremities. To set the TOT task, we used ADOC (21, 22), which allows therapist and patient to share goals. For example, the patient can select activities, which she or he would like to achieve, from the selection screen presented when making a choice and prioritizing activities. The patient would be able to imagine scenes using her or his paralyzed upper extremity in daily life, because ADOC presents many targeted activities with illustrations; even when there is difficulty with speech, the patient can communicate by pointing. Then, patient and therapist together make decisions on up to 5 important occupations and rate their extent of importance—the scale ranges from 1 (not important) to 4 (very important).

### 2.4 Outcome measures

In this pilot study, we selected several measures to explore the outcomes of task-oriented RFE.

The Fugl-Meyer Assessment (FMA) was used for evaluating motor control (29). FMA for the upper extremity consists of 4 components: A. shoulder / elbow / forearm (18 tasks), B. wrist joint (5 tasks), C. hand (7 tasks), and D. coordination / speed (3 tasks), for a total 33 tasks. Each task is evaluated on a scale with 3 grades (0: cannot perform the action, 1: can perform the action only partially, and 2: can perform the action fully), and the total score ranges from 0 (severe) to 66 (mild).

The Action Research Arm Test (ARAT) was used to assess the ability to manipulate objects (30). There are 19 sub-items within the four major items of grasp., grip, pinch, and gross movement. Each of these is rated on a four-point scale (0 to 3), with the total score ranging from 0 (severe) to 57 (mild).

The motor activity log (MAL) is a patient reported outcome used to measure the amount of use (AOU) and quality of movement (QOM) of the paralyzed hand in 14 daily living tasks (31). The MAL-AOU takes values from 0 (never used) to 5 (used often), and the MAL-QOM takes values from 0 (very poorly used) to 5 (used as well as pre-stroke).

The ADOC measured the goal to be achieved after 6 weeks on a scale of 1 (very dissatisfied) to 5 (very satisfied) (21).

All of the measures were taken before the start of treatment and again after 6 weeks of treatment. Evaluations were conducted by the individual therapist in charge, and the collected data were managed by one occupational therapist (KF).

To assess the feasibility of task-oriented-RFE, therapists recorded attendance and acceptability for therapy sessions, therapy content, duration (start and end times), and any adverse effects of the intervention. The clinical records were retrospectively reviewed by KF, including ensuring that each intervention adhered to the protocol.

### 2.5 Responses to adverse events

We paid special attention to adverse events that were expected: blood pressure fluctuation, muscle fatigue, and the appearance of pain, especially in the shoulder joint. If an adverse event occurred, the attending occupational therapist would have recorded it in the medical record. If pain persisted, the occupational therapist would have reported it to the attending physician and would have stopped the examination or training. If pain appeared during electrical stimulation, the intensity of the stimulation would have been adjusted. In addition, if a serious adverse event such as a fall had occurred, it would have been immediately reported to the attending physician.

### 2.6 Statistical analysis

FMA, ARAT, MAL-AOU, MAL-QOM, and ADOC were assessed before and immediately after completion of the 6-week intervention. To statistically compare measures taken before and after intervention, we calculated the difference between values at 6 weeks and baseline and estimated 95% confidence intervals (CI) by bootstrapping with 1,000 iterations for each of the measures (32, 33), which is recommended for small sample sizes. Measurements of all variables are summarized as mean and standard deviation (SD) or 95% CI. Statistical analyses were performed with IBM SPSS Statistics version 29.0 (International Business Machines Corp. Armonk, NY).

## 3 Results

Eight of 74 inpatients satisfied the inclusion criteria and agreed to join the current study. All 8 participants successfully completed the 6-week task-oriented RFE program, consisting of 80-min sessions per day and totaling 30 sessions, without any nonattendance, break requests, or refusal of training. There were no expected or unexpected adverse events during this study. All therapists adhered strictly to the protocol.

Changes in outcome measures for individual participants and summaries, including results of statistical analyses, are shown in Table 2. Confidence intervals for the means of the differences between 6-week and baseline values do not include zero, and the lower bounds of confidence intervals and the mean differences themselves are all far from zero, implying that the observed improvements in the outcome measures with therapy are real, not merely the result of chance.

**Table 2:** Individual changes in outcome measures with task-oriented RFE.

Patient		A	B	C	D	E	F	G	H	Mean (SD or 95% bootstrap CI)	*p* value*
FMA	Baseline	20	15	31	34	31	41	40	17	28.6 (10.1)	0.009
	Week 6	34	28	53	45	43	53	60	30	43.3 (11.8)	
	Difference	14	13	22	11	12	12	20	13	14.6 (12.1 to 17.6)	
ARAT	Baseline	10	6	9	26	13	30	32	11	17.1 (10.4)	0.06
	Week 6	21	11	41	39	22	40	57	24	31.9 (14.9)	
	Difference	11	5	32	13	9	10	25	13	14.8 (9.3 to 21.5)	
MAL-A	Baseline	0.5	0.23	0.33	0.3	0.25	1.16	1.55	0	0.54 (0.53)	0.014
	Week 6	3	1.15	3.83	1.76	1.91	2.5	3.05	1	2.28 (0.99)	
	Difference	2.5	0.92	3.5	1.46	1.66	1.34	1.5	1	1.74 (1.25 to 2.33)	
MAL-Q	Baseline	0.6	0.15	0.33	0.38	0.16	1	1.75	0	0.55 (0.58)	0.004
	Week 6	2.83	1.07	3.5	1.84	2.08	2.83	2.75	1.25	2.27 (0.85)	
	Difference	2.23	0.92	3.17	1.46	1.92	1.83	1	1.25	1.72 (1.27 to 2.24)	
ADOC	Baseline	1.5	1	1.25	1.5	1.66	1.33	1.5	1.5	1.41 (0.21)	0.001
	Week 6	3.5	3	4.25	3.5	3.66	3.66	4	3.5	3.63 (0.37)	
	Difference	2	2	3	2	2	2.33	2.5	2	2.23 (2.04 to 2.50)	

CI, confidence interval; FMA, Fugl-Meyer assessment; ARAT, action research arm test; MAL-A, motor activity log - amount of use; MAL-Q, motor activity log - quality of movement; ADOC, aid for decision-making in occupation choice. *p values in comparisons between values at 6 weeks and baseline in each group, according to the bootstrap method.

The mean change in FMA was 14.6 (SD 4.1, range 11–22) points; this exceeds both the minimal clinically important difference (MCID: 10 points) (34) and the minimal detectable change (MDC: 5.2 points) (35). The mean change in ARAT was 14.8 (SD 9.1, range 5–32) points; this exceeds the MCID for the dominant hand (12 points) but not that for the non-dominant hand (17 points) (36). The mean change in MAL-AOU was 1.74 (SD 0.86, range 0.92–3.50) points; this exceeds both the MCID (0.5 points) (37) and the MDC (0.84 points) (38). The mean change in MAL-QOM was 1.72 (SD 0.74, range 0.92–3.17) points; this exceeds both the MDC (0.77 points) (35) and the MCID (1.1 points) (39). The mean change in ADOC was 2.23 (SD 0.37, range 2.00–3.00) points; this exceeds the MDC (1.8 to 2.3 points) (40).

## 4 Discussion

This feasibility study demonstrated the following three points. First, the task-oriented RFE program as a combination therapy combining RFE and TOT is safe, well-tolerated, and feasible for patients with hemiplegic upper extremity after subacute stroke. Second, task-oriented RFE might lead to favorable outcomes in FMA, ARAT, and MAL scores and in the ADOC scale. Finally, ADOC might be useful in task or goal setting for task-oriented RFE.

All eight patients in this study completed, without dropout or adverse events, a 6-week, 80 min/day intervention (within the limit of what is covered by the Japanese health insurance system) during the convalescent phase. The mean age was 73.6, ranging from 47 to 91. Furthermore, task-oriented RFE was feasible regardless of whether paralysis, ranging from moderate to severe, was on the left or right side and whether it affected the dominant or non-dominant hand. This suggests that it could be applicable to a wide range of degree of paralysis and is convenient for patient recruitment. In addition, the intervention and evaluation could be implemented as originally planned. All upper extremity motor measures, including patient satisfaction (MAL or ADOC), presented favorable outcomes close to or exceeding their corresponding MDCs and MCIDs. These preliminary results suggest that the present treatment regimen is practical for treatment of patients with hemiplegic upper extremity. In a future randomized controlled trial adopting any of the current outcome measures, task-oriented RFE could be compared with other interventions in a real-world setting involving 80 min a day of daily occupational therapy in convalescent wards.

In the task-oriented RFE program, all patients underwent RFE-under-cNMES and TOT. Allocation of treatment time to each component can be adjusted from 20 to 60 min according to the decision of the occupational therapist, depending on both the degree of severity of paralysis and achievement of the task adjusted for difficulty. That is, if the paralysis is mild, one would shorten the RFE treatment time (by reducing the RFE pattern or number of repetitions) and lengthen the time of more practical TOT. Conversely, if the paralysis is severe, it is possible to alter the program, such as increasing the duration of RFE, which enhances the voluntarism of the affected upper extremity, or shortening the duration of “difficult” TOT so that the patient does not tire or so as to not induce compensatory movements. The total session length of 80 min was also based on the fact that the daily total maximum training time in which therapists are directly involved in the convalescent phase for a patient is limited to 180 min, including physical therapy and speech language therapy in addition to occupational therapy.

On the contrary, traditional CIMT is a well-evaluated exercise therapy but requires a higher dose of TOT (i.e., 6 h/d for 5d/wk. for 2 weeks); in addition, it requires that a mitten or glove be worn on the less impaired upper extremity and requires use of the paretic upper extremity for 90% of waking hours (18). In Japan, adherence to CIMT was only 35% in a rehabilitation educational hospital certified by the Japanese Association of Rehabilitation Medicine, despite its being highly recommended in the Japanese Guidelines for the Management of Stroke 2015 (24). On the other hand, a beneficial effect of CIMT can be obtained even if intervention time is reduced (0.5 h/d for 3d/wk. for 10 week) and upper extremity restraint on the non-paralyzed side is reduced to 5 h, which is called the modified version of CIMT (mCIMT) (41–43). According to an international survey (44), CIMT is used worldwide and about 75% is performed as mCIMT. For example, the elements of CIMT used in practice are “intensive graded task specific practice” (88.8% of 169 respondents) and “a transfer package” (38.5% of 169 respondent). The typical length of a daily therapy session during the CIMT program ranged from 0.5 h/d to 8 h/d, and the most commonly used duration was 1 h (30.2%), followed by 4 h, 6 h, and 2 h (about 10% each).

It has been reported that there is no significant correlation between intensity (i.e., time dosage or total rehabilitation time) of mCIMT and gain of motor recovery in patients with mild to moderate paralysis who showed improvement with a 2-week mCIMT [pre-intervention FMA, 45.1 (SD 12.4)] (45). On the other hand, TOT (1 h /d for 4d/wk. for 8 weeks, 4 different dose groups) for outpatients with mild-to-moderate chronic stroke paralysis has also been reported to show no significant dose related increase in physical performance in the paralytic upper extremity, when measured with an accelerometer during 24 h a day of daily activities (46). In addition, a TOT program of 1 h/d for 3d/wk. for 10 weeks in outpatients with moderate impairment after subacute stroke showed no significant difference with an equivalent or lower dose of usual occupational therapy for upper-extremity rehabilitation (47). Therefore, with either CIMT or TOT, an additional different type of therapy might be necessary, especially to achieve improvement with moderate or greater paralysis (48).

Recently, robotic therapy has been introduced and its efficacy has been reported in cases where patients presented with moderate to severe paralysis with FMA <30 during the convalescent period (49). Furthermore, case–control studies have reported favorable results with TOT with robotic therapy combined (50).

In addition, we envisage RFE as a candidate for effective exercise therapy when combined with TOT. In previous studies of an RFE program in the convalescent period, 40 min/d of RFE was combined with 30 min/d of dexterity-related or object-related training for the affected upper extremity (8, 10). On the other hand, the combination of CIMT and neurodevelopmental techniques, i.e., stretching, shaking, and weight bearing, was reported for 6 patients with chronic moderate to severe paralysis [mean baseline score of FMA, 24 (SD 7)] (25). In that study, the techniques were used especially when the frequent repetition of movement during some CIMT exercises tended to increase tonus in the affected upper extremity. Although systematic data were not shown, the techniques seemed to “result in improved posture, decreased synergic movement patterns, and improved tone in the trunk,” according to the therapist’s opinion. In contrast, the current task-oriented RFE program introduced the RFE session immediately before the TOT session within the daily session because RFE alone not only improves motor control but also might reduce spasticity (51).

Previous studies concerned with RFE have not reported outcomes related to real world use of the affected upper extremity. In the present study, however, increases of the outcomes were observed in all of the following areas: upper extremity and hand function, ability to manipulate objects, frequency and ease of use of the paralyzed hand, and satisfaction with the achievement of goals. Because this pilot study during the convalescent phase was conducted without a control intervention and was based on a small number of patients, we interpret the outcomes obtained on the basis of clinical meaningfulness rather than strict statistical testing. Because all measures showed improvements close to or exceeding their corresponding MDCs and MCIDs, the current Task-oriented RFE program seems to have the potential to lead to favorable outcomes regarding motor impairment, motor function, amount of use or quality of movement in daily use of the hemiplegic upper extremity, and satisfaction with goal setting.

We consider the strength of task-oriented RFE to be the synergistic effect of combining RFE and TOT, as explained in the following two points. One pertains to expanding the indication of TOT to patients who present with severe paralysis; the other pertains to more advanced task setting.

First, the combination of RFE may expand the indication of TOT to patients who present with severe paralysis. TOT is considered to be difficult to apply for moderate to severe upper extremity paralysis (47). On the other hand, RFE-under-cNMES has been reported to have efficacy in severe paralysis with FMA ≤ 20 (10), or Finger-Function items score in the Stroke Impairment Assessment Set of 0 or 1a (11), before the intervention. In this study, task-oriented RFE produced improved outcomes relative to MCID with FMA and MAL-AOU in patients with severe paralysis with FMA ≤ 20 (Patients A, B, and H). It is thought that even patients with severe paralysis, for whom TOT was otherwise difficult to perform, were able to undergo TOT because RFE-under-cNMES made it easier to move the paralyzed upper extremity, and this may have led to the favorable outcomes.

Second, improvement of motor control (i.e., facilitating isolated movement with the paralyzed upper limb/hand) with RFE-under-cNMES may allow for more advanced tasks during TOT. This may have made it possible to set a more practical task during TOT. In addition, RFE has finger-specific techniques, i.e., specialized therapeutic patterns for isolation of paretic fingers, that are rare in other manual therapies (8, 26). The combination of these strengths may have resulted in further improved outcomes regarding motor impairment or functions and enabled TOT with more advanced tasks, which may have contributed to more frequent use of the paralyzed hand and greater satisfaction with goal setting.

In the current task-oriented RFE program, we introduced ADOC not only measuring the goal to be achieved but also with a goal or task setting tool. The ADOC allows the patient and therapist to set and share specific goals (21). This might contribute to the application of improved object manipulation to increase use of the hand not only in the training session but also in real daily activities. We believe that fully assessing the intended use of the paralyzed upper extremity makes it easier to move the paralyzed hand and to use it in daily life, and might help prevent ‘learned non-use’. The increase in ADOC scale over the MDC suggests that task-oriented RFE is also effective in achieving the subject’s goals.

This pilot and feasibility study has several limitations. The sample size of 8 patients was too small to allow precise estimation of expected outcomes, and the interventions were performed during the subacute phase of stroke where spontaneous recovery could occur without even a control intervention. In addition, we did not record the number of minutes therapists performed RFE and TOT. Measurement duration of time spent on RFE and TOT per session or throughout the intervention might support developing a more detailed manualized procedure for administration of the intervention. Finally, it is especially unclear whether the combined RFE and TOT program is more effective than either program alone. Therefore, a future study needs to examine the effects under a randomized controlled trial design with appropriate sample size, participant-selection criteria, and comparison groups. Nevertheless, the present results suggest that such a trial would be justified.

## 5 Conclusion

This study was conducted to examine the feasibility of combining task-oriented RFE with RFE-under-cNMES and TOT for treatment, during the convalescent period, of patients with upper-extremity paralysis due to stroke. There were no expected or unexpected adverse events during the study period, and the task-oriented RFE program could be successfully carried out within 80 min a day of occupational therapy, which means also within the procedural constraints of the Japanese health insurance system during the convalescent phase. Mean changes in FMA, ARAT in the dominant hand, MAL-AOU, and MAL-QOM were greater than the minimal clinically important differences. Mean changes in ADOC were greater than the minimal detectable change. The favorable outcomes achieved by patients undergoing this treatment suggest that it is feasible and beneficial for recovery of upper-extremity paralysis in addition to increased use of the paralyzed hand in daily life. Further studies are warranted to examine the efficacy of combined RFE-under-cNMES and TOT.

## Data availability statement

The original contributions presented in the study are included in the article/supplementary material, further inquiries can be directed to the corresponding author.

## Ethics statement

The studies involving humans were approved by Ethics committee of Kirishima Sugiyasu Hospital. The studies were conducted in accordance with the local legislation and institutional requirements. The participants provided their written informed consent to participate in this study. Written informed consent was obtained from the individual(s) for the publication of any potentially identifiable images or data included in this article.

## Author contributions

KF: Conceptualization, Formal analysis, Writing – review & editing, Data curation, Investigation, Methodology, Project administration, Resources, Writing – original draft. MU: Conceptualization, Writing – review & editing, Investigation, Validation. SE: Conceptualization, Writing – review & editing, Formal analysis, Methodology, Validation. MS: Conceptualization, Formal analysis, Funding acquisition, Methodology, Supervision, Writing – review & editing, Validation, Writing – original draft.
